# Screening for pulmonary hypertension secondary to pulmonary tuberculosis

**DOI:** 10.7196/AJTCCM.2020.v26i4.129

**Published:** 2020-12-01

**Authors:** Richard Raine

**Affiliations:** Respiratory Clinic, Department of Medicine, Faculty of Health Sciences, University of Cape Town, South Africa

**Keywords:** Pulmonary tuberculosis, Pulmonary hypertension, Epidemiology, Screening

## Editorial

It is likely that some patients who have had pulmonary tuberculosis (PTB) will go on to develop pulmonary hypertension (PH). PTB affects many compartments of the lung with injury to airways, parenchyma and loss of pulmonary vascular bed. The possible association between PTB and PH has been suggested by several authors.^[1,2]^ To date, however, there has been a paucity of data confirming this suspicion, with a study^[3]^ from India showing that 15% of patients with PH had evidence of previous PTB.

This issue of the *AJTCCM* contains the results of a pilot study of patients with previously treated PTB who were evaluated for the presence of PH.^[4]^ The 20 patients included were screened for many other recognised causes of PH, and subjected to clinical, electrocardiographical, radiological, pulmonary function and echocardiographical testing. None of the patients had symptoms of dyspnoea, and there were no clinical findings suggestive of PH. Approximately one-third had electrocardiographical changes, and 85% had at least one chest radiographical feature compatible with PH. Pulmonary function was within normal limits. All patients underwent echocardiography and none of them were found to have elevated pulmonary artery pressures.

This small pilot study does not exclude the possibility of PH as a consequence of PTB, but raises the question of which patients, of the many who have had PTB, should undergo further evaluation. Most algorithms for screening for PH approach this issue by suggesting clinical suspicion, followed by echocardiography.^[5–7]^ Dyspnoea is the most prominent symptom in PH^[8]^ and the absence of this, perhaps, explains the results of this pilot study. Clinical examination, electrocardiography and chest radiology are likely to be helpful in more severe disease. Chest radiology with enlargement of the pulmonary artery associated with right ventricular enlargement, or peripheral pruning, had a high sensitivity and specificity for PH in patients with a 50% pre-test probability of PH.^[9]^ Kalla *et al*. ^[4]^ describe enlarged pulmonary arteries in 75% and pruning in 25% of patients, but do not mention whether these were combined. Echocardiography remains the most helpful screening tool, with chest computed tomography, ventilation-perfusion scans and right heart catheterisation remaining essential investigations once the diagnosis of PH is likely.^[5]^

PH secondary to PTB is likely to be multifactorial in origin. The prevalence remains unknown, and further studies to ascertain the true prevalence remain important. As a start, future studies should use symptoms, particularly dyspnoea, as an initial screening test to enhance detection.
